# Restoration of Leg Function with a Femoral Branch Nerve Transfer to the Anterior Obturator Nerve after an Iatrogenic Injury

**DOI:** 10.1055/a-2977-6940

**Published:** 2026-10-28

**Authors:** Julian Nachtsheim, Guido Beldi, Dietmar Bignion, Silvan Pasquinelli, Esther Vögelin

**Affiliations:** 1 Department of Plastic and Hand SurgeryInselspital, University Hospital BernBernSwitzerland; 2 Department of Orthopaedic Surgery and TraumatologyInselspital, University Hospital BernBernSwitzerland; 3 Department of Visceral Surgery and MedicineInselspital, University Hospital BernBernSwitzerland; 4 Department of Hand SurgeryOrthopädie Sonnenhof BernBernSwitzerland

**Keywords:** obturator nerve injury, peripheral nerve surgery, nerve transfer, hernia repair complication, nerve trauma

## Abstract

**Background:**

Iatrogenic injury of the obturator nerve represents an uncommon yet clinically relevant complication after pelvic surgery. If not recognized intraoperatively, conservative management remains the primary therapeutic approach. Surgical intervention may be indicated in cases with persistent symptoms. Although nerve transfer procedures are frequently used in nerve lesions of the upper extremity, they have rarely been employed for nerve injuries in the lower extremities.

**Case Presentation:**

We present the case of a 61-year-old patient who presented to our outpatient clinic 4 months after bilateral endoscopic obturator hernia surgery with significant weakness in right leg adduction, without any improvement through physiotherapy. After failed conservative therapy, he underwent an end-to-side transfer of a femoral branch to the obturator nerve to restore adduction of the thigh. Nine months after surgery, the patient was pain-free, showed good function of thigh adduction, was able to fully work as a metal worker, and could pursue his leisure sport activities without impairment. Electroneuromyography demonstrated that the right adductor magnus muscle was primarily innervated by the femoral nerve. No donor site morbidity was observed.

**Conclusion:**

Nerve transfer may therefore represent a valuable treatment option for patients with obturator nerve injury who do not show sufficient improvement with conservative management.

## Introduction

The obturator nerve, which arises from the rami of the second to fourth lumbar nerves, travels from the lumbar plexus along the psoas muscle, crosses over the obturator artery, and enters the medial thigh through the superior aspect of the obturator foramen. It enters the medial thigh through the obturator canal and divides into the anterior (innervates adductor brevis, adductor longus, gracilis, and may contribute to the innervation of the pectineus muscle) and posterior (innervates obturator externus and adductor magnus) division. Sensory function covers a small area of skin on the medial thigh overlying the adductor muscles. 1 2 Furthermore, it contains proprioceptive fibers for the hip and knee joints. 3

Obturator nerve injury is a significant complication that can arise after various gynecological and visceral pelvic surgeries, such as hernia repair. 4 5 6 7 The mechanisms of such injuries include nerve transection, clipping or electrocoagulation of the nerve, compression due to surgical mesh, or inadvertent traction during surgical manipulation. 8 9

Patients with obturator nerve damage present with adductor weakness, gait disturbances, and sensory deficits localized to the medial thigh. 4 10 Symptoms can result in a complex clinical picture that may be mistaken for other conditions and may lead to permanent neurological deficits. 4 11 12 Conservative treatment can lead to gradual symptom resolution in some cases and is therefore usually the first treatment option when iatrogenic nerve injury is not recognized during surgery. 10 13 At our institution, we do not adhere to a period of watchful waiting when a nerve was demonstrably functional before and non-functional after a given procedure. In such cases, we perform surgical exploration with intraoperative direct nerve stimulation within 4 weeks of the index operation to assess continuity and conduction of the nerve. This deviates from the commonly recommended interval of 3 to 4 months, 14 and is based on the observation that retrograde neuronal loss begins within weeks of complete transection and that delayed referral remains the most important modifiable determinant of outcome. 14 15 If the injury is recognized during the index procedure, direct end-to-end repair is occasionally feasible. In most cases, however, a nerve defect is present, and reconstruction with autologous nerve grafts (sural nerve grafts), 16 17 allogeneic nerve grafts, 18 artificial nerve conduits, 5 19 20 or nerve transfers (end-to-end or end-to-side) from femoral branches 21 is recommended.

To the best of our knowledge, the literature on reconstruction or repair of the obturator nerve following iatrogenic injury consists only of case reports. Regarding the use of a motor branch transfer of the femoral nerve to the obturator nerve, there is only one previously published case report. 21

## Case Presentation

We present a 61-year-old patient with right obturator neuropathy following bilateral endoscopic repair of obturator hernias, first seen in our outpatient clinic 4 months after surgery. After the procedure, the patient experienced sensory deficits caudal and medial to the inguinal ligament and on the medial thigh. In addition, significant weakness in right leg adduction (0/5), using the Medical Research Council (MRC) grading system to assess muscle strength, was present. The patient did not show any improvement through physiotherapy. He reported unsteadiness while walking, could not pursue his leisure activities such as badminton, and experienced a significantly reduced quality of life.

Electroneuromyography confirmed denervation with spontaneous activity and an absence of voluntary activation of the adductor longus muscle. Magnetic resonance neurography showed hyperintense signal changes and thickening with potential signs of compression of the obturator nerve, as well as hyperintense T2 signal in the obturator-innervated muscles of the right thigh ( Fig. 1 ).

**Fig. 1:**
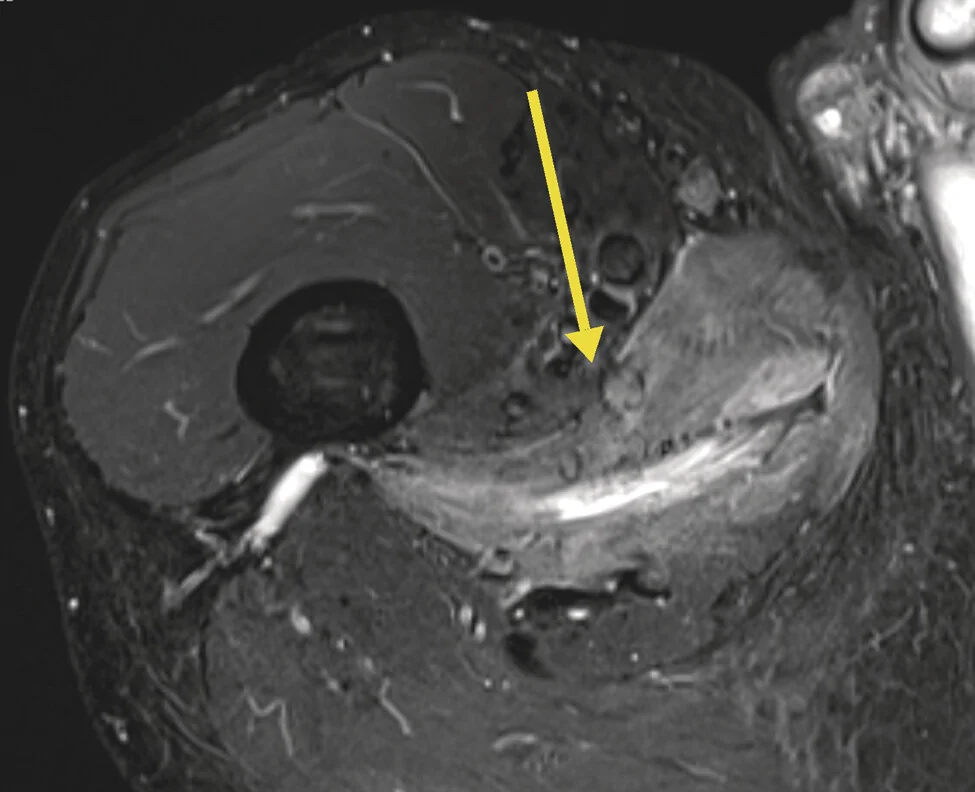
Axial T2-weighted TIRM right thigh: hyperintense signal of the obturator-innervated muscles, consistent with denervation edema (arrow). TIRM, turbo inversion recovery magnitude.

After another 3 months of conservative therapy with targeted physiotherapy had failed, revision surgery was performed 7 months after the hernia repair, following the recommendations described by Johnson et al. 22

In collaboration with the Visceral Surgery team, an ilioinguinal approach was performed with the patient in a supine position, following preoperative sonographic localization of the obturator nerve. After preservation and neurolysis of the genitofemoral nerve, the abdominal wall musculature was divided, and the iliopsoas muscle along with the external femoral vessels was exposed retroperitoneally. Under protection of the right ureter subsequently, the obturator nerve was exposed superior and proximal to the obturator foramen. Here, the mesh was found to be firmly adherent to the nerve ( Fig. 2 ). After partial resection of the mesh, complete microsurgical neurolysis was performed. Nerve continuity was confirmed; however, the obturator nerve appeared markedly thickened and scarred, suggestive of a Sunderland grade III lesion ( Fig. 3 ). Intraoperative nerve stimulation did not show any motor response.

**Fig. 2:**
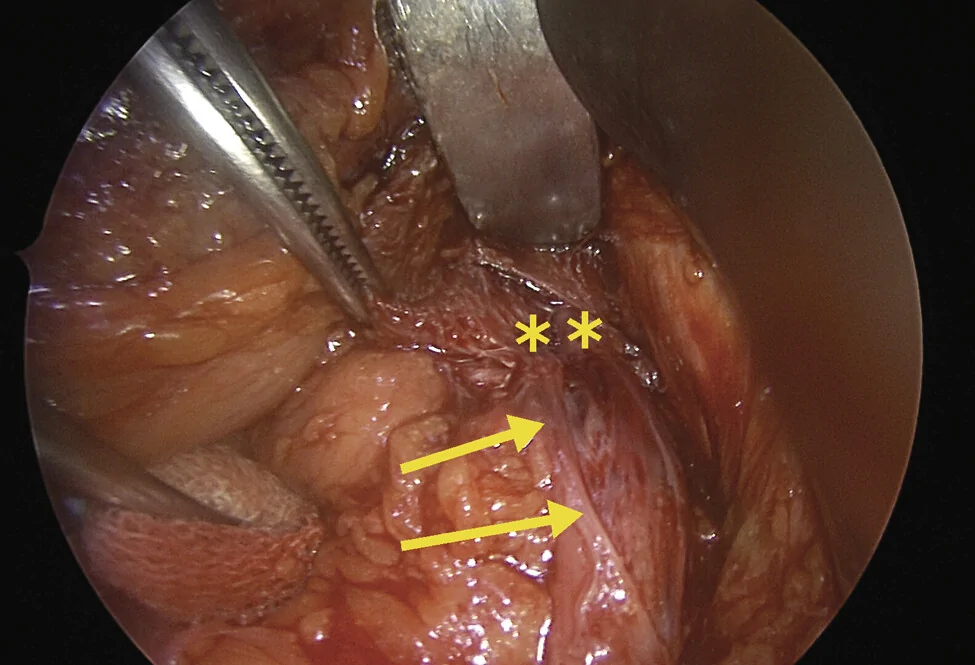
Intraoperative view showing the caudal portion of the mesh adherent to the obturator nerve (asterisks), before neurolysis and partial resection of the mesh. The arrows indicate the obturator nerve.

**Fig. 3:**
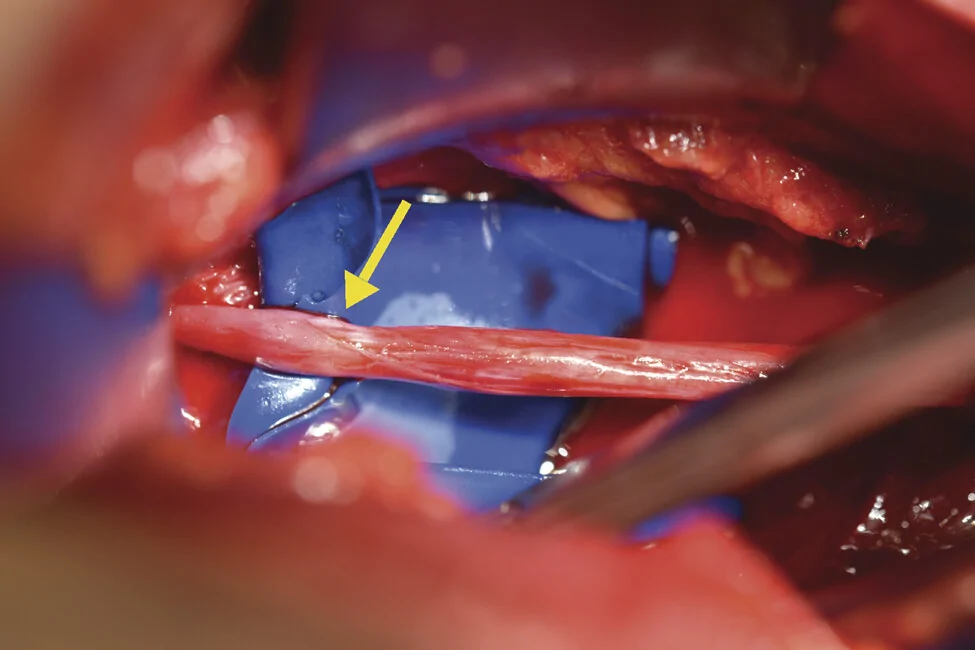
Obturator nerve after removal of the caudal portion of the mesh. The nerve is thickened, hardened, and constricted (arrow), suggestive of a Sunderland grade III lesion.

Due to the traction injury, the nerve was distally exposed. A V-shaped incision was made on the proximal medial thigh (~ 8 cm distal to the inguinal ligament). The femoral neurovascular bundle was exposed, and dissection proceeded medially toward the adductor brevis and adductor longus muscles. The anterior division of the obturator nerve was ultimately identified at the posteromedial border of the adductor longus muscle. After exposure of the femoral nerve, three motor nerve branches to the sartorius muscle were identified. One of these branches was transected as distally as possible and passed beneath the adductor longus muscle to the anterior division of the obturator nerve. The transferred fascicle was microsurgically coapted end-to-side in a tension-free manner using two epineural 8–0 nylon sutures ( Fig. 4 ). Due to the traction injury, it was decided to perform an end-to-side and not an end-to-end nerve transfer. Finally, fibrin sealant (Tisseel; Baxter Healthcare Corporation) was applied.

**Fig. 4:**
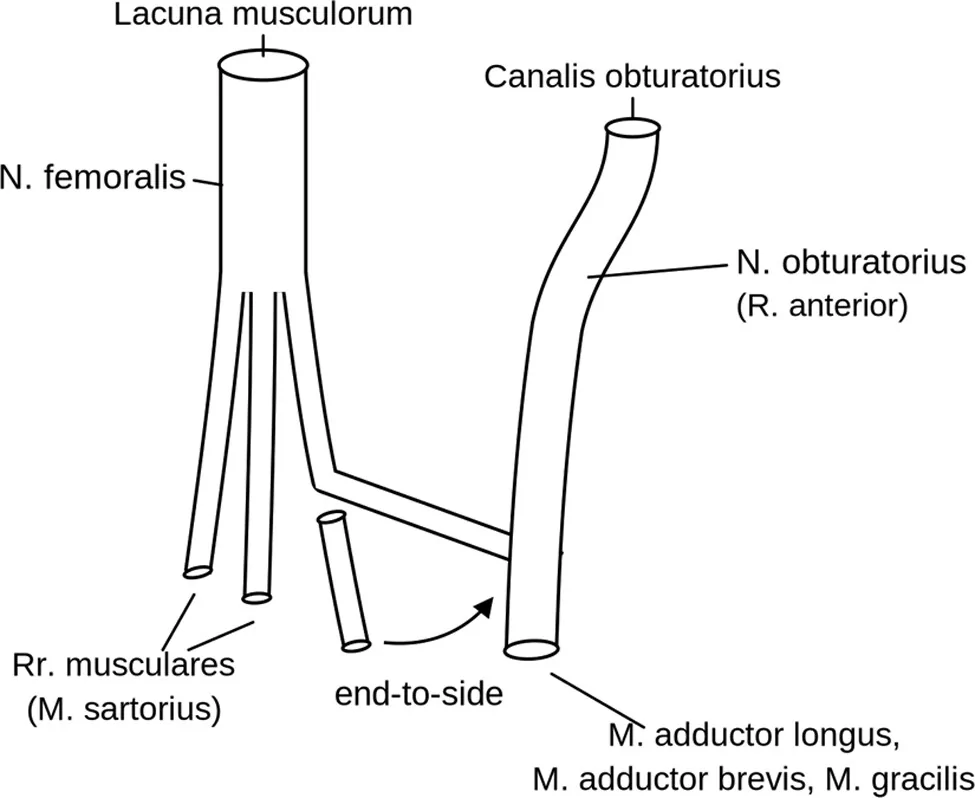
Schematic illustration of the end-to-side transfer of a motor branch of the femoral nerve to the anterior division of the obturator nerve. One of the three motor branches to the sartorius muscle was transected as distally as possible; the distal stump is shown below the coaptation site. The arrow indicates the direction of transfer.

Postoperatively, a compression bandage was applied. Full weight-bearing was permitted, without restrictions on range of motion.

The patient was seen 6 months postoperatively in the outpatient clinic, demonstrating good recovery. He had resumed badminton and fitness training and had returned to work as a metal worker. Clinically, he was able to walk without limping. The leg adduction could be forcefully performed (MRC 4/5, Fig. 5 ). He displayed a physiological gait pattern and was completely pain-free. Sonography revealed superficial denervation edema of the gracilis muscle, while the other adductor muscles showed normal fascicular structure ( Fig. 6 ). No donor site morbidity of the taken nerve branches had been observed.

**Fig. 5:**
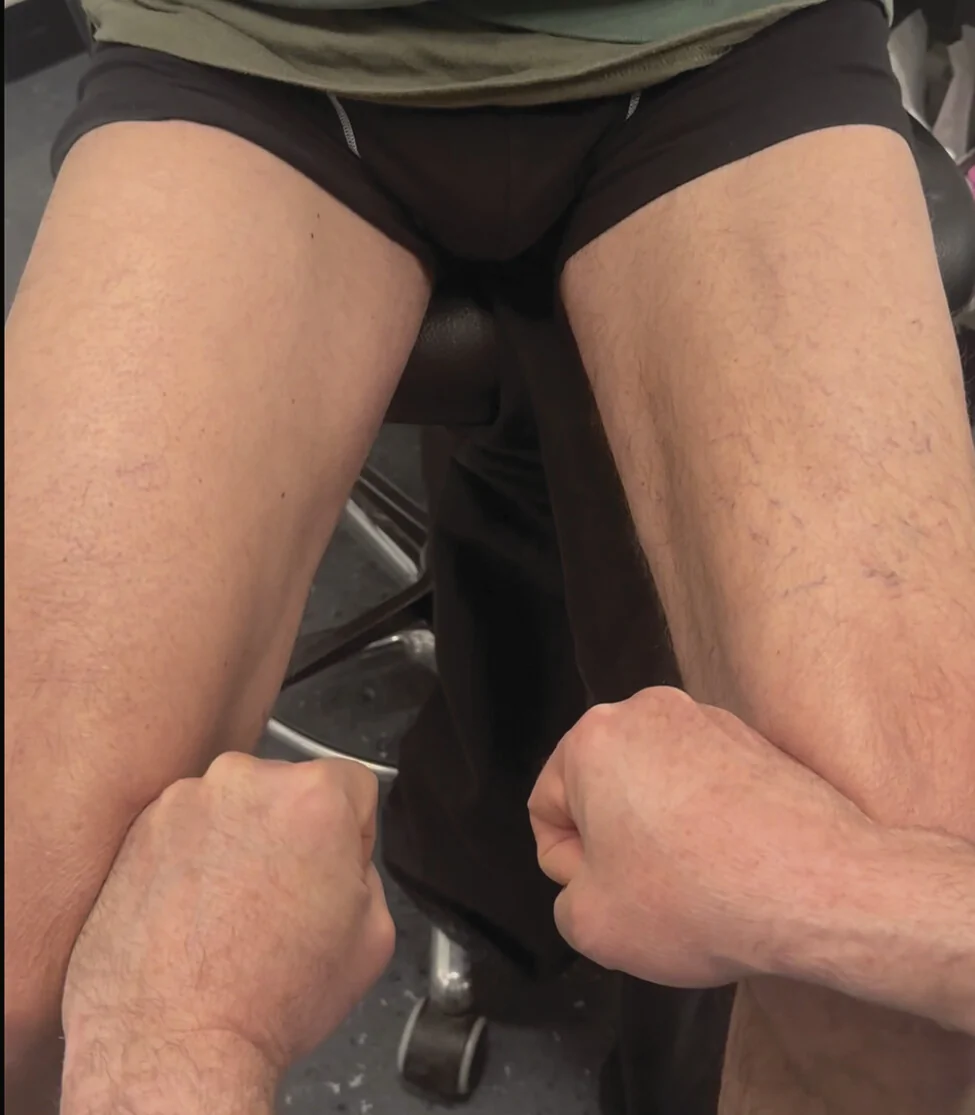
Hip adduction against resistance 6 months postoperatively (Medical Research Council grade 4/5).

**Fig. 6:**
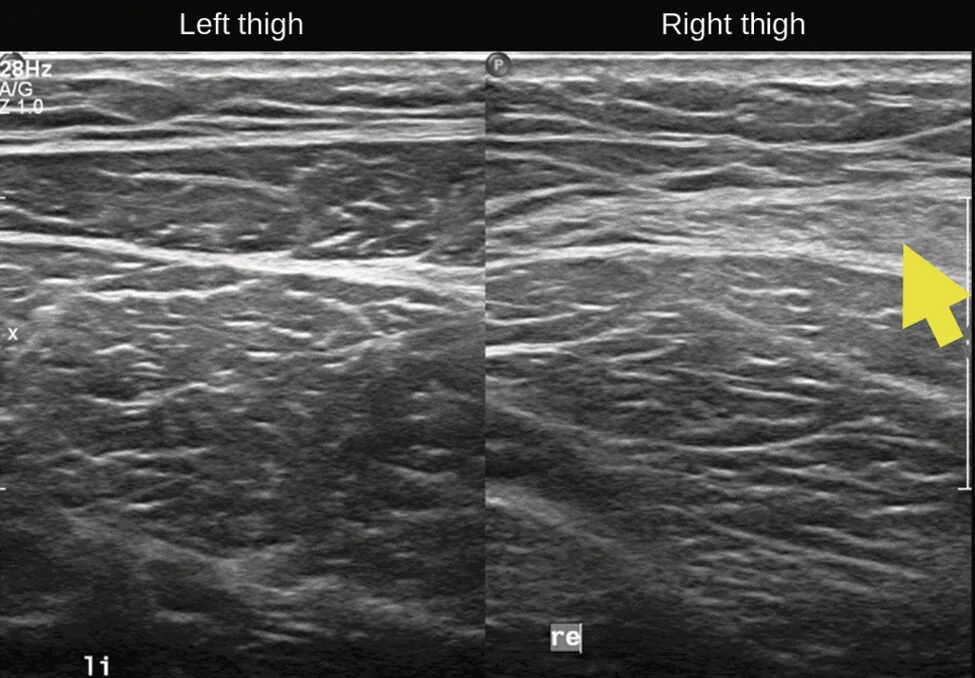
Ultrasound of the adductor muscles 6 months after surgery. Superficial denervation edema of the right gracilis muscle (arrow); the remaining adductor muscles show a normal fascicular structure.

Nine months after surgery, electrophysiological examination demonstrated reinnervation of the right adductor magnus muscle mainly by the femoral nerve. After stimulation of the obturator nerve, no compound muscle action potential could be recorded from the adductor muscles ( Table 1 ).

**Table 1:** Electrophysiological findings 9 months postoperatively

EMG nerve	Muscle	Latency (ms)	Amplitude (mV)	Time (ms)
Right femoral nerve	Right adductor magnus	4.9	4.2	9.6
Right obturator nerve	Right adductor magnus	None	None	None
Right femoral nerve	Vastus medialis	12.9	2.2	4.2
Right obturator nerve	Vastus medialis	None	None	None

Abbreviation: EMG, electromyography.

## Discussion

Obturator nerve injury is an infrequent but potentially severe complication in gynecological surgery—especially in cases of genitourinary malignancies—as well as in urological or visceral pelvic surgery. 4 5 17 Various treatment options have been described. Most case reports focus on direct nerve repair, usually resulting in complete resolution of symptoms within 1 year. 5 17 20 23 24 There is very little literature addressing chronic obturator nerve lesions and the options for secondary reconstruction.

If the nerve injury is identified immediately during primary surgery, direct end-to-end neurorrhaphy or reconstruction with an artificial nerve conduit, an autologous nerve graft, or a nerve allograft appears to be a feasible treatment option. In their case report, Andan et al reported excellent outcomes with complete clinical recovery 6 months after direct end-to-end neurorrhaphy. 5 Trapasso et al described three cases in which intraoperatively identified obturator nerve injuries during surgical treatment of gynecologic malignancies with lymph node dissections were managed with direct end-to-end repair, resulting in satisfactory functional recovery of the adductor muscles. 20 Matsumoto et al also reported excellent function 15 months after immediate use of an artificial nerve conduit. 19 Harma et al described an intraoperatively recognized obturator nerve injury, which was repaired using an autologous nerve graft from the ipsilateral sural nerve, with additional application of fibrin sealant (Tisseel; Baxter Healthcare Corporation). The patient achieved restoration of adduction from 2/5 to full strength (5/5) within 6 months postoperatively. 17

Ghaemmaghami et al and Beneš described immediate sural nerve grafting with microsurgical sutures without fibrin glue; both reported good functional outcomes. 23 24 Dias et al also reported immediate 3 cm sural nerve cable grafting, using three grafts placed side by side. Six months postoperatively, complete recovery of leg adduction was achieved. A clear advantage of using three grafts side by side could not be demonstrated in comparison to the other case reports. 16

To date, the only authors who have described an approach comparable to ours are Spiliopoulos and Williams. They reported the case of a 56-year-old woman with an obturator nerve injury following total abdominal hysterectomy and bilateral salpingo-oophorectomy, in addition to right external iliac and obturator lymph node dissection for endometrial and ovarian adenocarcinoma. Seven months after the primary surgery, they performed a femoral branch-to-obturator nerve transfer distal to the obturator foramen, as further intrapelvic exploration was considered to pose a high intraoperative risk due to postoperative adhesions and altered anatomy after the oncologic procedure. The patient experienced full recovery of adductor strength within 10 months, accompanied by improvement of the sensory deficit over the medial thigh. 21

## Conclusion

In chronic, symptomatic obturator nerve lesions, femoral nerve branch transfer and neurolysis represent a viable option. Although different techniques have been described, surgical management of chronic postoperative obturator nerve lesions is rarely reported. Nerve transfer using one of the three femoral nerve branches to the sartorius muscle seems to be a feasible treatment option without donor site morbidity.

## Declaration of GenAI Use

During the preparation of this manuscript the authors used a generative AI assistant (Claude, Anthropic) for language editing. No content, data, or references were generated by the tool. The authors reviewed and edited all output and take full responsibility for the content of the publication.
